# Continuing to evolve: opportunities to share technology enhancements with health sciences library peers through the Virtual Projects Section

**DOI:** 10.5195/jmla.2023.1824

**Published:** 2023-10-02

**Authors:** Emily J. Hurst

**Affiliations:** 1 ejhurst@vcu.edu, Virtual Projects Advisory Committee, Journal of the Medical Library Association, and Deputy Director/Head, Research and Education, Health Sciences Library, Virginia Commonwealth University Libraries, Richmond, VA.

## Abstract

Beginning in 2012, the Virtual Projects section of the *Journal of the Medical Library Association* has provided an opportunity for library leaders and technology experts to share with others how new technologies are being adopted by health sciences libraries. From educational purposes to online tools that enhance library services or access to resources, the Virtual Projects section brings technology use examples to the forefront. Virtual Projects highlighted in this year's section include new ways to use virtual reality for library instruction, podcasting to share important health care messages with the Latino Community, enhancing findability by using options in a library management system, and developing a research profiling system. After a hiatus due to publishing changes in 2022, 2023 will bring some major changes for the section. The new publication issue for future Virtual Projects sections will be January and the call for submissions and Virtual Projects deadline will now take place in June and July.

Technology enhancements are often at the heart of today's health sciences libraries. Innovative librarians and professional staff have long embraced opportunities to use technology to advance library services and spaces. Technology use in the classroom has changed health sciences education. Technology options to better connect hybrid and remote staff impact daily work in libraries. Technology solutions range from vendor-generated options that have been customized to meet the unique needs of a library to home-grown projects that require knowledge of coding or the application of technology tools in new or innovative ways.

While technology use and projects in health sciences libraries have become commonplace, the use of the Virtual Projects section continues to provide a publication opportunity dedicated to sharing these case examples in the hopes of showcasing technology innovation in libraries and helping identify technology leaders and potential colleagues for collaboration. In an effort to increase and encourage submissions for section consideration, authors of contributed content submissions for the Medical Library Association Annual Meeting/Conference are invited to self-select or opt-in to having their abstract considered for selection in the *JMLA* Virtual Projects section. The number of projects reaching the Virtual Project Advisory Committee committee have increased in range and scope since this option was added in 2020.

Membership demographics in the Medical Library Association (MLA) continue to change and the Virtual Project Advisory Committee acknowledges that more technology showcases are necessary to continue to raise awareness of the Virtual Projects section. The current Virtual Projects Advisory Committee is considering new options to further promote the section and attract even more submissions in the future. One of the major shifts will be moving the Virtual Projects section from the October *JMLA* issue to the January issue, beginning in 2024. We hope this will allow more time for the Virtual Projects Advisory Committee to reach out to individuals directly to encourage submissions.

Although selected for submissions in 2022, technologies including podcasting, virtual reality, enhancements for library management systems, and iresearcher profiling systems are all still relevant in today's health sciences library setting. This year's section addresses a range of technology topics with unique uses in health sciences settings:

- JUNTOS Radio: a podcast created in collaboration with Spanish-speaking healthcare providers, Juntos Center for Advancing Latino Health, and a medical librarian
- Providing health sciences education through virtual reality experiences
- From Digital Commons to Alma/Primo: enhancing the dissemination of Doctor of Nursing Practice (DNP) projects
- Researcher profiling systems: fostering collaboration on a regional medical campus and clinical and translational science award institution

Material for this year's column was selected in 2022 with the assistance of the *JMLA* Virtual Projects Advisory Committee: Christine Andresen; Emily J. Hurst section editor; Michelle Kraft, AHIP, section coeditor; J. Dale Prince, AHIP; Tariq Rahaman; and Brian Zelip. Selected projects were edited by Emily J. Hurst.

